# Sensitive and Specific Detection of SARS-CoV-2 Antibodies Using a High-Throughput, Fully Automated Liquid-Handling Robotic System

**DOI:** 10.1177/2472630320950663

**Published:** 2020-12

**Authors:** Donna Grace Karp, Deanne Cuda, Devangkumar Tandel, Kenneth Danh, Peter V. Robinson, David Seftel, Honglin Tian, Mark Pandori, Kevin W. P. Miller, Cheng-T. Tsai

**Affiliations:** 1 Enable Biosciences, South San Francisco, CA, USA; 2 Nevada State Public Health Laboratory, Reno, NV, USA; 3 Hamilton Company, Reno, NV, USA

**Keywords:** PCR, antibody, agglutination, liquid handling, anti–spike protein

## Abstract

As of July 22, 2020, more than 14.7 million infections of SARS-CoV-2, the virus responsible for Coronavirus Disease 2019 (COVID-19), have been confirmed globally. Serological assays are essential for community screening, assessing infection prevalence, aiding identification of infected patients, and enacting appropriate treatment and quarantine protocols in the battle against this rapidly expanding pandemic. Antibody detection by agglutination–PCR (ADAP) is a pure solution phase immunoassay that generates a PCR amplifiable signal when patient antibodies agglutinate DNA-barcoded antigen probes into a dense immune complex. Here, we present an ultrasensitive and high-throughput automated liquid biopsy assay based on the Hamilton Microlab ADAP STAR automated liquid-handling platform, which was developed and validated for the qualitative detection of total antibodies against spike protein 1 (S1) of SARS-CoV-2 that uses as little as 4 µL of serum. To assess the clinical performance of the ADAP assay, 57 PCR-confirmed COVID-19 patients and 223 control patients were tested. The assay showed a sensitivity of 98% (56/57) and a specificity of 99.55% (222/223). Notably, the SARS-CoV-2–negative control patients included individuals with other common coronaviral infections, such as CoV-NL63 and CoV-HKU, which did not cross-react. In addition to high performance, the hands-free automated workstation enabled high-throughput sample processing to reduce screening workload while helping to minimize analyst contact with biohazardous samples. Therefore, the ADAP STAR liquid-handling workstation can be used as a valuable tool to address the COVID-19 global pandemic.

## Introduction

The ongoing coronavirus disease 2019 (COVID-19) pandemic is caused by the severe acute respiratory syndrome coronavirus 2 (SARS-CoV-2), which has infected many millions of individuals and claimed hundreds of thousands of lives throughout 195 countries/regions.^1,2^ These numbers continue to rise. The clinical manifestations and severity of COVID-19 patients vary widely, from asymptomatic to fever, fatigue, cough, rashes, coagulopathy, and life-threatening multiorgan failure.^3^

Serological testing complements direct pathogen detection by identifying diagnostically confirmative host immune responses. The presence of antibodies against SARS-CoV-2 indicates recent or past exposure to the pathogen.^4,5^ Studies have shown that SARS-CoV-2 antibodies are detectable in most patients 2 weeks after the onset of symptoms and that anti–spike protein S1 subunit antibody signals are positively correlated with neutralization assay results.^5^ These advancements have positioned serological testing as a vital method for tracing infection chains, surveying epidemiological prevalence of COVID-19 within communities, identifying convalescent blood donors, and monitoring antibody response in vaccine testing. Some current work is also being performed to investigate the persistence of antibodies after infection and vaccination.^6^

**Figure 1.**
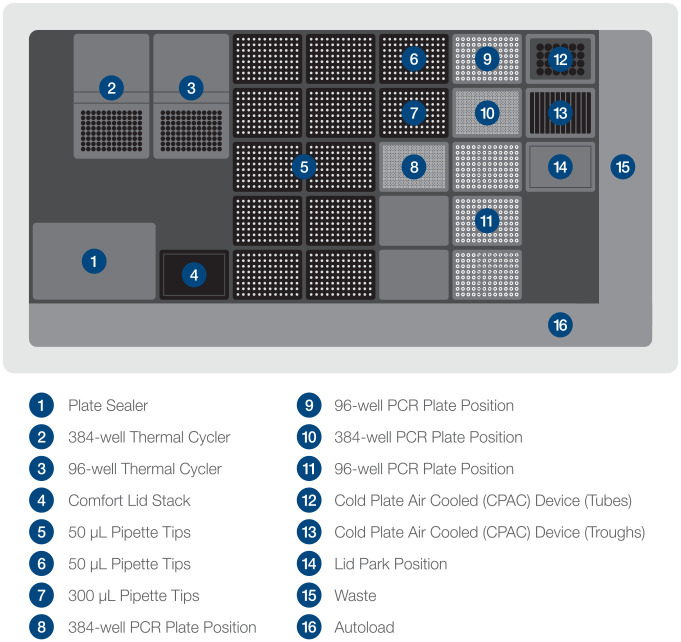
Instrument deck layout, based on the Microlab ADAP STAR.

In this study, we report the development and validation of a highly sensitive and specific SARS-CoV-2 total antibody assay on a Hamilton MicroLab STAR liquid-handling platform (**Fig. 1**), based on the ADAP STAR assay-ready workstation. The assay is based on antibody detection by agglutination-PCR (ADAP) technology, which has been previously applied to a variety of autoimmune and infectious diseases with high performance.^7–10^ The highly sensitive nature of the ADAP assay enables a substantial reduction in the required sample size, which helps facilitate testing in populations in which large-volume blood draws are onerous or unpalatable. With ADAP, two or more antibody-antigen binding events are required before a valid signal is generated, and this confers greatly improved specificity to the assay. Automation of the assay is readily achieved owing to ADAP’s operationally simple workflow (e.g., no washing or centrifugation steps). The successful implementation of the automated high-throughput ADAP SARS-CoV-2 total antibody assay solution as described herein can help meet the surge in demand for COVID-19 infection testing.

## Materials and Methods

### Materials

Consumables used in the study included 96- and 384-well PCR frame plates (P/Ns 814302, 814305), 50 µL nonsterile conductive filter tips (P/N 235948), 300 µL nonsterile conductive filter tips (P/N 235903), PCR Comfort Lids (P/N 814300), and Optically Clear Plate Seals (P/N 67765-01), all from Hamilton Company (Reno, NV), together with 12-column reagent reservoirs from Agilent Technologies (Santa Clara, CA; P/N 201256-100). ADAP SARS-CoV-2 Total Antibody Assay reagent kits were manufactured by Enable Biosciences (P/N DK2-100 [SARS-CoV-2]). The SARS-CoV-2 spike protein S1 #REC31806-100) was purchased in >90% purity from the Native Antigen Company (Oxford, UK). Molecular biology grade water (P/N 46-000-CM) was purchased from Corning (Tewksbury, MA). Chemicals were purchased from Sigma-Aldrich (St. Louis, MO), unless otherwise stated.

### Human Specimens

Blood specimens used in this study were obtained from various sources. These included remnant serum specimens from RT-PCR–confirmed COVID-19 patients, obtained from the California Department of Public Health and the Nevada Department of Public Health and sourced from discarded clinical laboratory specimens exempted from informed consent and institutional review board (IRB) approval under the condition of patient anonymity. Three convalescent COVID-19 patients donated serum samples with informed consent under Enable Biosciences IRB #20180015 (approved by Western IRB). Serum samples collected from blood donors with other viral or bacterial infection prior to the outbreak of COVID-19 (*n* = 119) were purchased from commercial biobanks. Remnant serum samples collected from RT-PCR–negative individuals during the outbreak (*n* = 24) were obtained from the California Department of Public Health. All specimens collected outside of the Enable Biosciences clinical network were received as de-identified specimens.

### Instrumentation and Accessories

A Hamilton Microlab STAR with a deck layout based on the ADAP STAR assay-ready workstation and containing eight motorized independent pipette channels, autoload barcode reader, Hamilton plate sealer, Inheco on deck thermal cycler (ODTC), and Inheco cold plate air-cooled device on deck was used to execute all automated workflows. The instrument was operated by Hamilton Venus software with total aspiration and dispense monitoring features enabled for all pipetting actions. The Bio-Rad CFX384 Touch real-time PCR detection system was used for assay readout.

### Antibody Assay

The ADAP SARS-CoV-2 antibody assay (DK2-100, Enable Biosciences, South San Francisco, CA) was composed of conjugate mixtures (containing a pair of SARS-CoV-2-DNA conjugates and buffers), ligation mixture (made of DNA ligase, bridge oligonucleotide), preamplification mixture (made of primers, polymerase), and qPCR mixture (made of primers and SYBR master mix). The assay used buffer C (made of phosphate-buffered saline and Triton-X) as blank controls. The positive controls were made by diluting stock SARS-CoV-2 antibodies into serum matrix, whereas the negative controls were made of non–COVID-19 serum.

Patient samples were collected via standard serum tubes or serum separation tubes, from which a serum component separated within 24 h of collection. The serum samples were then stored at either 2 to 8 °C for 1 week or −80 °C for longer periods until the ADAP assay was able to be performed. Each assay consumed 4 µL of the serum sample without the need for further processing.

Each analysis entailed the use of test specimens, buffer C blank controls, positive controls, and negative controls. The test specimens were analyzed in their own individual well. Buffer C blank controls were analyzed in 8 replicates to establish the background CT values, and positive and negative controls were run in duplicate to ensure the quality of the plate. Plate data were accepted only if both replicates of the positive and negative controls were deemed positive and negative, respectively.

Briefly, agglutination mix (8 µL) and the appropriate body fluid sample (4 µL) were added to a 384-well frame plate, transferred to the 384-well ODTC, and incubated at 37 °C for 30 min. Then, the resulting mix (4 µL) and ligation mix (116 µL) were added to a 96-well frame plate, transferred to the 96-well ODTC, and incubated at 30 °C for 15 min. Next, that resulting mix (25 µL) and preamplification mix (25 µL) were added to a 96-well frame plate, transferred to the 96-well ODTC, and subjected to 13 cycles of PCR (cycling between 95 °C and 56 ° C for a total of approximately 40 min). The amplified product was diluted 20-fold using molecular biology grade water. Finally, the diluted product (8.5 µL) was added to each individual qPCR-primer mix (11.5 µL). This final solution was sealed using the on-deck plate sealer for subsequent qPCR quantification on a CFX384 Touch Real-Time Detection System (Bio-Rad Laboratories, Hercules, CA).

The assay readout ΔC_T_ is defined as the difference in C_T_ values between the average of eight buffer C blank controls and the samples as previously described. The value of ΔC_T_ is proportional to the initial amplicon concentrations in the PCR plate well. This amplicon concentration is also proportional to the amount of target antibodies present in the samples.

The manual analyses of ADAP were conducted using the same protocol (e.g., reagent volumes, incubation temperature, and duration) with the exception that the pipetting was done manually, the incubation and thermal cycling steps were performed on a Bio-Rad 96-well deep-well thermal cycler, and the quantification was performed on a CFX96 Touch Real-Time Detection System (Bio-Rad Laboratories). The automated platform was not used at all in manual processes.

### Data Analysis

PRISM v8.1.1 and XLSTAT software 2019.1 were used for data analysis. A *p* value with an alpha of 0.05 was used as the cutoff for significance.

## Results

### Principle of the ADAP SARS-CoV-2 Total Antibody Assay

**Figure 2.**
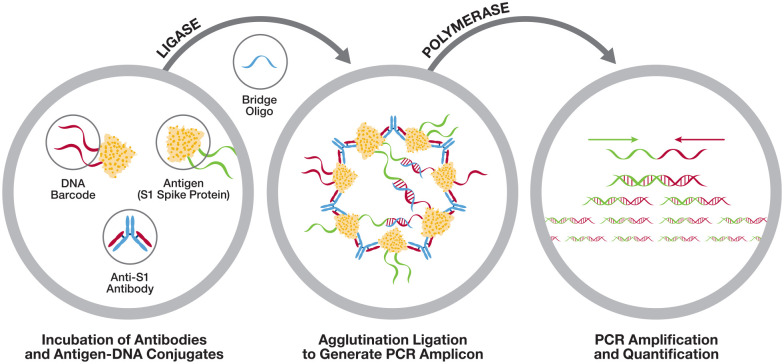
Antibody detection by agglutination (ADAP) differs from traditional immunoassays in that it is a pure solution-phase assay and is well-suited to detecting sensitive antibodies. The automated SARS-CoV-2 total antibody assay on the ADAP STAR is a completely hands-free workflow that uses a pair of antigen-DNA conjugates to probe target antibodies within a given sample (circle 1). If antibodies are present, then the antibody and antigen agglutinate, and close proximity allows the DNA conjugates to ligate (circle 2), leading to the formation of specific DNA amplicons that can then be quantified offline using qPCR (Circle 3).

The ADAP SARS-CoV-2 total antibody assay is based on the ADAP technology, whose use was previously reported for infectious diseases such as HIV, metabolic disease such as type 1 diabetes, and allergy testing. The general workflow is presented in **Figure 2**. To adapt the ADAP assay for the detection of COVID-19–related antibodies, a pair of DNA barcodes was installed on the SARS-CoV-2 spike protein S1 subunit. The S1 protein is a high-quality antigen in that it shares less homology with other coronaviruses (~64% with S1 protein from SARS 2003),^11^ whereas the nucleocapsid (N) protein shares extremely high homology (~91% with SARS 2003).^12^ Thus, our utilization of S1 protein was chosen to potentially render the assay highly specific with less cross-reactivity to other coronaviruses. Furthermore, several studies have shown that signals from anti-S1 protein antibodies are correlated with neutralization capability.^5,13^ In the past, coronaviral neutralization ability has been linked to protection for a period of time,^14^ and future studies may substantiate a similarity in COVID-19. Because of these favorable attributes, we chose to develop the ADAP SARS-CoV-2 total antibody assay based on the S1 protein.

The ADAP assay resembles a latex bead agglutination assay in that the anti–SARS-CoV-2 S1 protein antibodies in the sample can aggregate the S1 protein probes into a dense immune complex. Instead of using latex beads as reporters, however, the ADAP assay uses a pair of potentially complementary S1-single-stranded DNA conjugates. The proximal single-stranded DNA barcodes within the immune complex are brought close enough to each other such that the addition of a ligase and bridge oligonucleotide can reunite the two types of complementary DNA barcodes into a full-length amplicon suitable for successful PCR amplification and quantification. In the absence of the target antibodies, the two potentially complementary S1-DNA conjugates are too distant to be united. Each DNA strand carries only one primer binding site and is thus not individually amplifiable. This unique design enables a simple workflow in which unreacted probes do not have to be removed by washing or centrifugation. The quantity of the PCR amplicon is directly proportional to the quantity of antibodies in the specimen.

Notably, because the ADAP assay does not require a species- or isotype-specific secondary antibody for signal generation, the assay is capable of detecting all antibody isotypes/subtypes (e.g., immunoglobulin G, immunoglobulin M, immunoglobulin A) with two or more antigen-binding sites. The very same assay in principle is therefore also applicable to primate, rodent, and other species to facilitate antibody characterization in animal models, delivering important utility for vaccine research or zoological disease tracking.

### Sensitivity of ADAP Assay for SARS-CoV-2 Total Antibody in COVID-19 Patients

To evaluate the assay’s sensitivity, 57 serum specimens from COVID-19 patients were subjected to the ADAP SARS-CoV-2 total antibody analysis. These patients were confirmed to be COVID-19 infected based on positive RNA test results by RT-PCR. SARS-CoV-2 total antibodies were detected in 16 of 17 specimens collected within 15 days of symptom onset. In 40 specimens collected beyond 15 days of symptom onset, all were positive for SARS-CoV-2 total antibody. Thus, the assay had a sensitivity of 100% for patients 16 days after onset and an overall sensitivity of 98.25% for all COVID-19 patients across the entire disease presentation time frame (Table 1).

**Table 1.** The ADAP SARS-CoV-2 Total Antibody Assay Is Sensitive Even in the Early Phase of Infection.

Day after Symptom Onset	Number of Samples	Number of Reactives	Sensitivity (95% CI)
≤15 days	17	16	94% (69%–100%)
>15 days	40	40	100% (89%–100%)
Total	57	56	98% (89%–100%)

ADAP = antibody detection by agglutination; CI = confidence interval.

### Specificity of ADAP Assay for SARS-CoV-2 Total Antibody

To evaluate the assay’s specificity, 223 serum specimens (199 serum specimens collected prior to the outbreak and 24 specimens collected from RT-PCR–negative individuals during the outbreak) were tested for SARS-CoV-2 total antibody. The ADAP assay correctly identified 222 samples as being negative for the SARS-CoV-2 total antibody. The only positive specimen was from an individual with thyroid disease. The signal was 2.03, while the assay cutoff was 1.40. Thus, the assay had a specificity of 99.55% (**Table 2**).

**Table 2.** The ADAP SARS-CoV-2 Total Antibody Assay Is Highly Specific.

Total No. of Samples	No. of Nonreactives	Specificity (95% CI)
223	222	99.55% (97.14%–99.98%)

ADAP = antibody detection by agglutination; CI = confidence interval.

These control specimens included patients with infection and/or autoimmune diseases: 15 specimens positive for HIV antibodies, 6 specimens positive for hepatitis C virus (HCV) antibodies, and 10 specimens positive for anti-nuclear antibody (ANA). Notably, two samples from patients with PCR-confirmed NL63 coronavirus infection and one sample from individuals with HKU1 coronavirus infection were also included. These samples served as a powerful sample set to evaluate the cross-reactivity of the ADAP assay. Satisfactorily, all of these samples tested negative for SARS-CoV-2 total antibodies.

### Reproducibility of the ADAP Assay for SARS-CoV-2 Total Antibody

The within-run precision of the ADAP SARS-CoV-2 total antibody assay was evaluated by testing two positive specimens and one negative specimen 20 times in the same run (**Table 3**). The assay cutoff was 1.40, and all positive samples remained positive, whereas all negative samples remained negative. The between-run precision of the ADAP SARS-CoV-2 total antibody assay was evaluated by testing two positive specimens and one negative specimen five times on five separate runs on different days (**Table 4**). The negative and positive specimens consistently retained correct designations on each day. For positive specimens, the intra- and interassay variations ranged from 6.81% to 18.78%.

**Table 3.** Intra-Assay Variation of the Automated ADAP SARS-CoV-2 Total Antibody Assay (Assay Cutoff = 1.40).

Specimen	*n*	Mean (ΔC_T_)	SD	%CV
Negative 1	20	−0.41	0.32	N/A
Positive 1	20	2.07	0.37	17.69
Positive 2	20	4.25	0.29	6.81

ADAP = antibody detection by agglutination; CV = coefficient of variation.

**Table 4.** Inter-Assay Variation of the Automated ADAP SARS-CoV-2 Total Antibody Assay.

Specimen	*n*	Mean	SD	%CV
Negative 1	25	−0.18	0.15	N/A
Positive 1	25	1.82	0.23	12.91
Positive 2	25	3.87	0.73	18.78

ADAP = antibody detection by agglutination; CV = coefficient of variation.

### Manual and Automated Assay Correlation

**Figure 3.**
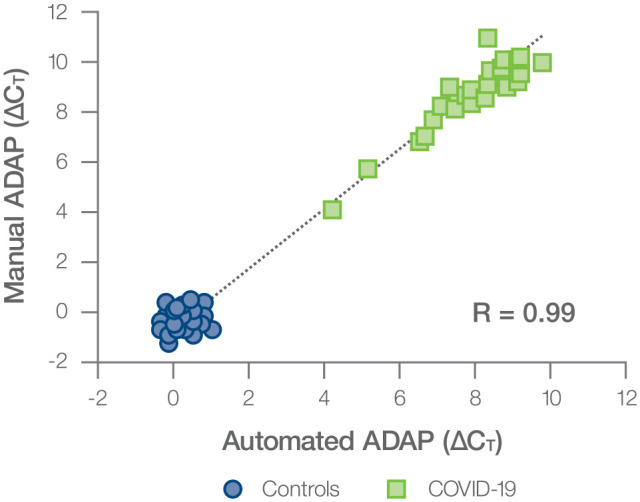
High correlation between the automated and manually performed antibody detection by agglutination assays. The assay cutoffs were 1.40 for both manual and automated methods.

**Figure 4.**
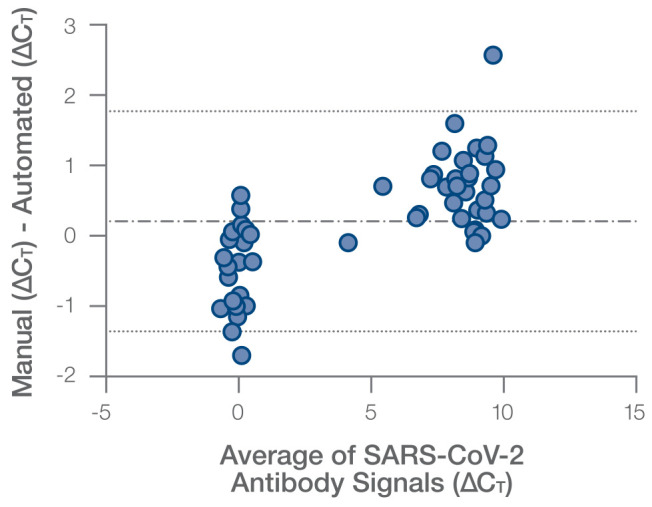
Bland-Altman plot to compare signals from the automated and manual antibody detection by agglutination assays. The middle dashed and dotted line is the mean difference, whereas the dotted lines to either side (top and bottom) mark the limits of two standard deviations from the mean.

To evaluate the signal correlation between manual and automated assays, we selected 18 COVID-19 patient samples and 24 control specimens with sufficient volumes for reanalysis by the manual method. The manual assay was strictly performed using the same procedure as the automated method, except that the pipetting was performed manually and the incubation and thermal cycling were performed on a Bio-Rad 96-deep-well PCR instrument. The result showed that the two methods were highly correlated, with a Pearson’s correlation coefficient *R* of 0.99 (**Fig. 3**). The assay cutoff was 1.40. All 18 COVID-19 patient samples tested positive and 24 control specimens tested negative by the manual method, indicating a 100% concordance with the automated analysis. To further analyze the difference between signals, we used a Bland-Altman plot to evaluate the two underlying methods (**Fig. 4**). The plot showed a mean difference (ΔCt) of 0.21, with an upper and lower limit of 1.96 standard deviations (ΔCt) at 1.78 and −1.36, respectively.

## Discussion

The SARS-CoV-2 pandemic created an unprecedented demand for accurate high-throughput clinical diagnostics. Reliable, automated analysis of SARS-CoV-2 is critically needed. In this study, we present a largely hands-free automated solution for the SARS-CoV-2 total antibody assay on the Hamilton MicroLab ADAP STAR system. This automated platform can greatly reduce labor requirements and enable a safer and more efficient laboratory workflow. Through an analysis of 57 COVID-19 and 223 control specimens, we showed that the automated system is highly sensitive (98%) and specific (99.55%).

Considering that the serological assay is often used in serosurveillance, it is of great interest to relay the ADAP assay sensitivity and specificity to positive predictive value (PPV) and negative predictive value (NPV). For instance, in a city with a moderate prevalence of COVID-19 infection at 5%, the PPV and NPV are 92.02% and 99.91%, respectively; whereas in a city with a high prevalence of COVID-19 infection at 10%, the PPV and NPV are 96.05% and 99.80%, respectively. These numbers support the utility of the ADAP assays in areas with moderate to strong exposure to SARS-CoV-2.

The ADAP assay format offers several advantages over traditional immunoassays such as enzyme-linked immunosorbent assay. First, each assay consumes only 4 µL of sample. The sample-sparing nature of the ADAP assay can facilitate sample analyses from those individuals for whom large-volume blood draws are difficult and preserve critical residual sample volume for other concordant analyses. Second, the ADAP assay is highly sensitive owing to the power of PCR amplification. Here, we demonstrated that the assay is capable of achieving 100% sensitivity for patients after 15 days of symptom onset. Third, the DNA barcoding attribute of ADAP makes it possible to include additional antigens in the assay to address distinct applications. For instance, several vaccine candidates that target the spike protein of SARS-CoV-2 have entered into various phases of human clinical trials.^15^ In the event that these vaccines are widely administered, the SARS-CoV-2 antibody assay against spike protein (e.g., S1 subunit, receptor binding domain) could be used to monitor the persistence of vaccine-induced antibodies, whereas further inclusion of nucleoprotein may be used to detect de novo immunity to actual infection.

We envision that the ADAP SARS-CoV-2 total antibody assay can used as a valuable serological tool to detect and quantify immune responses in COVID-19 patients. Although antibodies against SARS-COV-2 have not been explicitly shown as equivalent to immunity, the emerging evidence thus far is suggestive of some degree of immunity, and antibodies can clearly reveal recent or past exposure to the disease. Automated screening of antibodies on a large scale can also illuminate the incidence rate in a given population, which could be used to guide policies on disease control.^16^ Published studies have also demonstrated the important utility of antibodies in contact tracing.^17^

In summary, the ADAP SARS-CoV-2 total antibody assay is capable of detecting all antibody isotypes/subtypes in infected individuals and exhibits 100% sensitivity for the detection of the SARS-CoV-2 S1 protein 16 days after onset. The assay is highly sensitive and does not show cross-reactivity in samples infected with HIV, HCV, ANA, NL63, or HKU1. As expected, the results obtained from both the manual and automated workflows are concordant, with the automated platform exhibiting a high degree of reproducibility both within single assays and between assays run over time.
